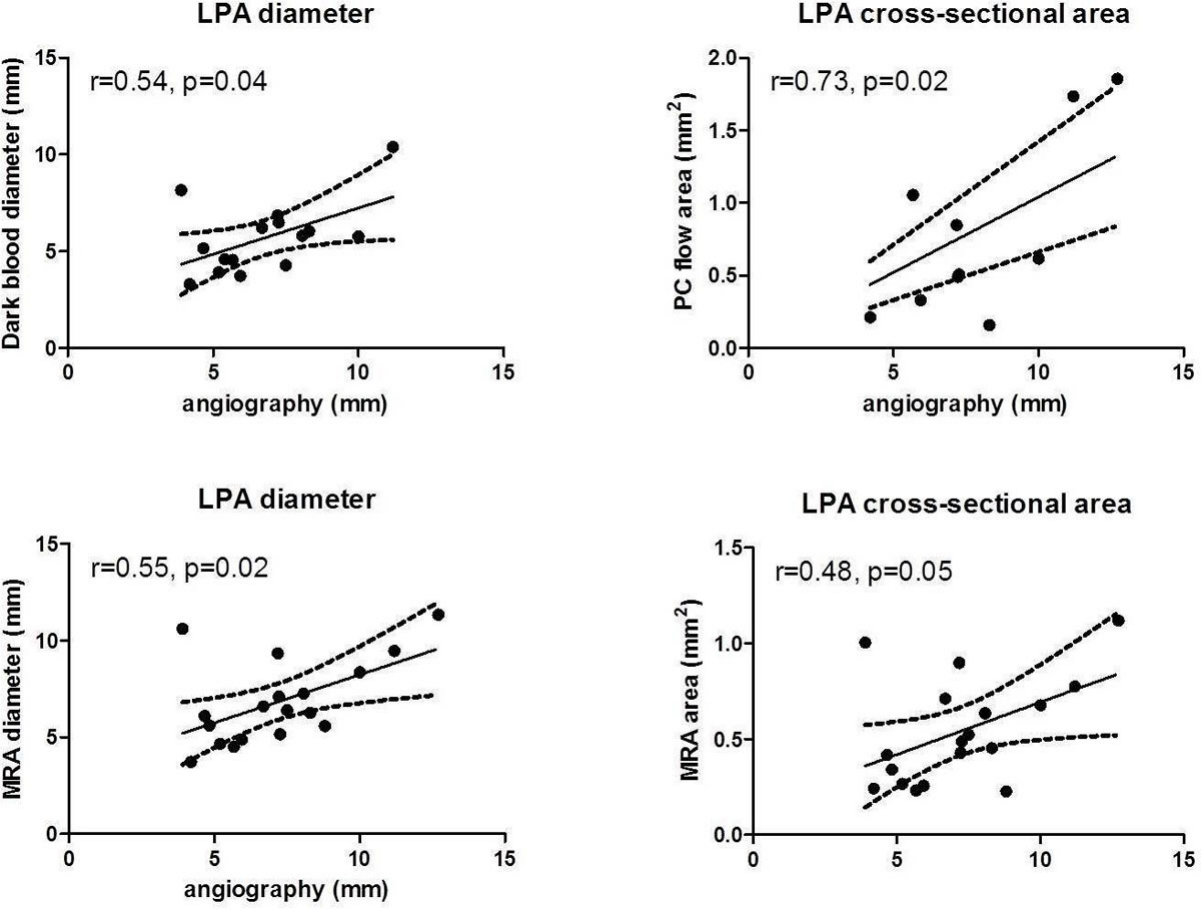# Which MRI sequence provides the most accurate estimate of branch pulmonary artery size in children with single ventricle?

**DOI:** 10.1186/1532-429X-15-S1-P295

**Published:** 2013-01-30

**Authors:** Edythe B Tham, Kimberley A Myers, Chodchanok Vijarnsorn, David J Patton, Michelle Noga

**Affiliations:** 1 Pediatric Cardiology, Stollery Children's Hospital, Edmonton, AB, Canada; 2 Pediatric Cardiology, Alberta Children's Hospital, Calgary, AB, Canada

## Background

Accurate evaluation of branch pulmonary artery size in children with single ventricle is important for surgical planning and has prognostic implications after Fontan surgery. Various cardiac MRI sequences are employed to evaluate these sometimes hypoplastic pulmonary arteries, which increases imaging time in anesthetised children. The aim of this study was to evaluate which MRI sequence(s) correlate with angiography measurements of pulmonary artery size made at cardiac catheterization in children with single ventricle.

## Methods

Twenty children (age: 21±30 months, weight 10±7kg) with single ventricle who underwent cardiac catheterization with pulmonary angiography, and cardiac MRI (Siemens 1.5T) within 43±29 days were included at any surgical stage. Pulmonary arteries were measured within the hilum, proximal to the first branch in both MRI and catheterization angiography. We used 4 different MRI sequences to assess pulmonary artery size: 1) Axial black blood (HASTE or TSE), 2) Axial cine, 3) Phase contrast (PC) through plane flow, and 4) 3D contrast-enhanced MRA images. The maximum diameter was measured in mid-systole for cines. For MRA images, the long axis of each pulmonary artery was reconstructed using multiplanar reformatting (MPR) and diameters in 2 dimensions were measured (RPA: axial and coronal, LPA: axial and sagittal-oblique). Cross-sectional areas and diameters were measured from MRA and through plane flow images. Pearson's correlation and linear regression with 95% confidence intervals were used to compare MRI with the "gold" standard catheterization angiography measurements (p<0.05).

## Results

For the RPA, all MRA measured dimensions correlated well with catheterization angiography (axial r=0.81, coronal r=0.89, cross-sectional area r=0.81, p<0.0001), with axial dark blood (r=0.65, p=0.02) and cine diameters (r=0.67, p=0.03) showing modest correlations. In contrast, LPA through plane PC area correlated best (r=0.73, p=0.02), with modest correlations for axial dark blood (r=0.54, p=0.04), MRA axial diameter (r=0.55, p=0.02), and MRA cross-sectional area (r=0.48, p=0.05).

## Conclusions

In children with single ventricle, MPR contrast enhanced MRA measurements showed the best correlate of RPA size compared to catheterization angiography. However, LPA cross-sectional area from PC flow measurements, and not MRA, were the best correlate of LPA measurements. Selection of the most appropriate sequence will provide a more accurate pre-operative evaluation of pulmonary artery size to aid the surgical strategy in this population. Knowledge of this will help tailor protocols to study pulmonary artery size in this population and imaging time may be shortened with this in mind.

## Funding

none

**Figure 1:**
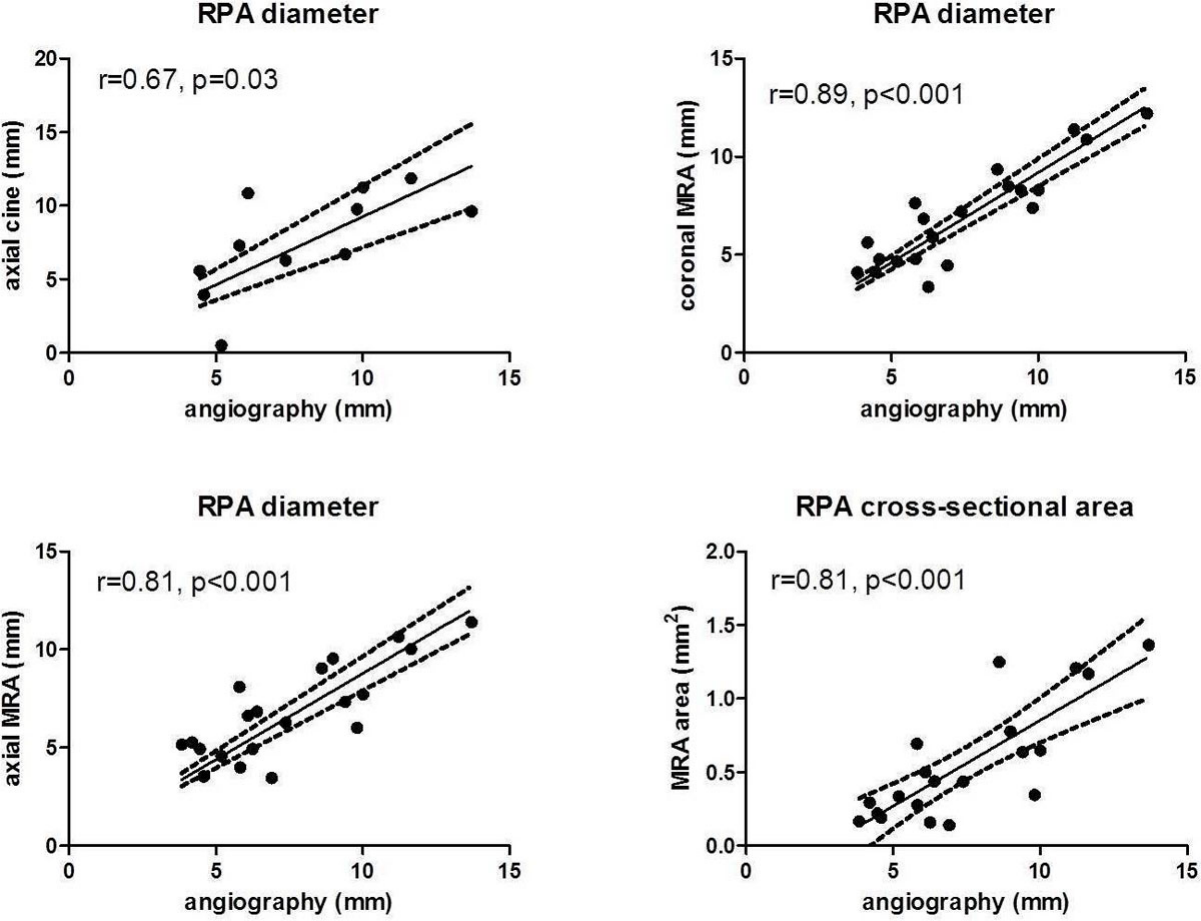


**Figure 2:**